# Genetic diversity among pandemic 2009 influenza viruses isolated from a transmission chain

**DOI:** 10.1186/1743-422X-10-116

**Published:** 2013-04-12

**Authors:** Sarah L Fordyce, Karoline Bragstad, Svend Stenvang Pedersen, Thøger G Jensen, Bente Gahrn-Hansen, Rod Daniels, Alan Hay, Marie-Louise Kampmann, Christian AW Bruhn, J Victor Moreno-Mayar, María C Ávila-Arcos, M Thomas P Gilbert, Lars P Nielsen

**Affiliations:** 1 Centre for GeoGenetics, Natural History Museum of Denmark, Øster Voldgade 5-7, Copenhagen K, 1350, Denmark; 2 Department of Virology, National Influenza Center, Statens Serum Institut, 5 Oerestads Boulevard 5, Copenhagen S, 2300, Denmark; 3 Department of Infectious Diseases, Odense University Hospital, 29 Sdr. Boulevard, Odense C, 5000, Denmark; 4 Department of Clinical Microbiology, Odense University Hospital, J.B.Winsløwsvej 21, 2, Odense C, 5000, Denmark; 5 WHO Collaborating Centre for Reference and Research on Influenza, MRC National Institute for Medical Research, London, UK

**Keywords:** Deep sequencing, Intra-host variation, Oseltamivir-resistance, A(H1N1)pdm09 influenza

## Abstract

**Background:**

Influenza viruses such as swine-origin influenza A(H1N1) virus (A(H1N1)pdm09) generate genetic diversity due to the high error rate of their RNA polymerase, often resulting in mixed genotype populations (intra-host variants) within a single infection. This variation helps influenza to rapidly respond to selection pressures, such as those imposed by the immunological host response and antiviral therapy. We have applied deep sequencing to characterize influenza intra-host variation in a transmission chain consisting of three cases due to oseltamivir-sensitive viruses, and one derived oseltamivir-resistant case.

**Methods:**

Following detection of the A(H1N1)pdm09 infections, we deep-sequenced the complete *NA* gene from two of the oseltamivir-sensitive virus-infected cases, and all eight gene segments of the viruses causing the remaining two cases.

**Results:**

No evidence for the resistance-causing mutation (resulting in *NA* H275Y substitution) was observed in the oseltamivir-sensitive cases. Furthermore, deep sequencing revealed a subpopulation of oseltamivir-sensitive viruses in the case carrying resistant viruses. We detected higher levels of intra-host variation in the case carrying oseltamivir-resistant viruses than in those infected with oseltamivir-sensitive viruses.

**Conclusions:**

Oseltamivir-resistance was only detected after prophylaxis with oseltamivir, suggesting that the mutation was selected for as a result of antiviral intervention. The persisting oseltamivir-sensitive virus population in the case carrying resistant viruses suggests either that a small proportion survive the treatment, or that the oseltamivir-sensitive virus rapidly re-establishes itself in the virus population after the bottleneck. Moreover, the increased intra-host variation in the oseltamivir-resistant case is consistent with the hypothesis that the population diversity of a RNA virus can increase rapidly following a population bottleneck.

## Background

Influenza A (family Orthomyxoviridae) is a negative sense, single-stranded RNA virus that infects a large variety of hosts, including aquatic birds, humans and swine. The influenza genome consists of eight segments, ranging from 890–2341 nucleotides (nt) in length, encoding up to 13 proteins. Reassortment of the eight segments between coinfecting strains results in genetic diversity of influenza virus. Such multiple-reassortment events occurring between numerous hosts were the cause of at least two pandemics in the 20^th^ century and the 2009 pandemic due to the triple-reassortant swine-origin A(H1N1)pdm09 virus [1].

In addition to reassortment, influenza viruses generate genetic diversity due to the high error rate of their RNA polymerase during transcription. This results in mixed populations of virus genotypes within a single infection (intra-host variants), that often exhibit varying levels of fitness [2]. Furthermore, the high mutation rate enables the virus to rapidly adapt to selective pressures, whether due to the host’s immune system or antiviral intervention. Genetic change can result in a virus subtype acquiring advantageous mutations such as those conferring antiviral-resistance. As the progeny of the successfully adapting viruses replace the other viruses in the population, population bottlenecks occur [3]. These profoundly affect the dynamics of virus evolution, and an improved understanding of these intra-host processes may assist in the development of more effective antiviral treatments.

Until recently, the genetic diversity of a virus population could only be assessed by reverse-transcriptase PCR (RT-PCR) [4], RT-PCR/electroscopy ionization mass spectrometry (ESI-MS) [5], or by isolating and cloning individual viruses and applying Sanger sequencing [6]. However, these approaches are time-consuming and laborious, hence only a few studies have analyzed influenza populations in great detail [7-11]. Deep sequencing techniques, including the Roche Genome Sequencer (GS) FLX and Illumina Genome Analyzer (GA) platforms, can detect low-frequency mutations and can provide considerable information on intra-host virus populations and the relative frequencies of virus variants. For instance, Ramakrishnan *et al*. [8] performed the first full influenza genome *de novo* Roche GS FLX sequencing for the purpose of identifying mixed infections and intra-host variation. Other studies have applied deep sequencing to patient samples from A(H1N1)pdm09-infected individuals to reveal intra-host diversity of the virus in relation to the presence of oseltamivir-resistance conferring mutations [7,9].

Here we present a case-study where the mode of A(H1N1)pdm09 influenza transmission between four infected individuals is known, with the emergence of oseltamivir-resistant A(H1N1)pdm09 viruses in the last of the cases (http://who.int/csr/disease/swineflu/notes/h1n1_antiviral_resistance_20090708/en/index.html).

Using a novel real-time RT-PCR assay to initially detect A(H1N1)pdm09, we subsequently applied deep sequencing techniques to investigate the intra-host diversity in the cases infected with oseltamivir-sensitive viruses compared to the case carrying oseltamivir-resistant viruses.

### The study

On 2 June 2009, Danish health authorities were notified that a Danish family (a mother and her two children, 11 and 12 years old) had been in close contact with a confirmed case of A(H1N1)pdm09 influenza infection during a flight from the USA to the Netherlands. At the time of notification, both children had influenza-like symptoms (cases A and B). Upon returning to Denmark, the family had close contact with seven people, one of whom developed influenza-like symptoms (sore throat, aching muscles, fever) (case C). Throat swabs were taken from all cases and contacts, and all were quarantined. Clinical cases were treated with oseltamivir, while contacts were given prophylactic doses of oseltamivir (see Table 1 for dosages). Cases A, B and C subsequently tested positive for oseltamivir-sensitive A(H1N1)pdm09 virus (described below), while the asymptomatic contacts were all influenza negative. Four days after initiation of oseltamivir prophylaxis, a second of the seven family contacts (case D) developed influenza-like illness (fever, sore throat, muscular pain) and tested positive for oseltamivir-resistant A(H1N1)pdm09 by real-time RT-PCR. Case D, who was in a close, personal relationship with case C, was previously healthy without underlying chronic disease. Contacts of case D were followed up but no cases due to transmission of resistant virus were found.

**Table 1:** Specimen collection and oseltamivir treatment information

**Patient**	**Date of specimen collection**	**A(H1N1)pdm + or -**	**Specimen type**	**Dose (mg)**	**Duration**
A	2 June 2009	+	Throat swab	60 twice daily	5 days
B	2 June 2009	+	Throat swab	60 twice daily	5 days
C	2 June 2009	+	Throat swab	75 twice daily	5 days
D	2 June 2009	-	Throat swab	75 once daily	5 days
	7 June 2009	+	Throat swab	None	N/A

**Footnotes:** + indicates patient testing positive for A(H1N1)pdm09 based on RT-PCR, whereas – denotes negative result.

## Results

Real-time RT-PCR assays confirmed the presence of A(H1N1)pdm09 in all four cases. Viruses recovered from case C remained sensitive to oseltamivir, however, A(H1N1)pdm09 viruses from case D exhibited a high level of resistance (see Table 2 for neuraminidase inhibitor test results). Viruses recovered from all cases remained sensitive to zanamivir (Table 2).

**Table 2:** Neuraminidase inhibition assay

**Samples**	**Oseltamivir IC**_50_**(nM)**	**Zanamivir IC**_50_**(nM)**	Fold difference compared to sensitive control* for Oseltamivir IC_**50**_
Sample C	4.5	2.5	3
Sample D	357	1.1	238
A/Norway/1758/07 (seasonal H1N1 sensitive control)	1.5	0.9	1
A/Norway/1735/07 (seasonal H1N1 resistant control)	494	0.8	329

**Footnotes**: *sensitive control refers to A/Norway/1758/07.

### Roche GS FLX sequencing of NA gene

We performed deep sequencing of the influenza *NA* gene from all cases (cases A and B were uncultured, whereas cases C and D were cell cultured), on the Roche GS FLX, and full influenza genomes from cases C and D, on the Illumina GAIIx, for the detection of intra-host variants. Roche GS FLX sequencing yielded full coverage of the *NA* genes from cases A, B, C and D with a respective average depth of coverage of 198, 110, 127 and 85. The oseltamivir-resistance causing mutation site, encoding residue 275 (N1 numbering), was covered by sequences at a depth of 228, 93, 84 and 132 for cases A, B, C and D, respectively. Cases A-C were homologous for encoded histidine. This provides evidence against the possibility that oseltamivir-resistant viruses were present at detectable levels within the virus populations in these 3 individuals. However, 86% of the sequences in case D (114/132) contained the resistance-conferring mutation (see Table 3). Furthermore, cases A-C showed no sign of intra-host variation at any locations throughout the *NA* gene and case D contained no other sites with intra-host variation other than the *NA* H275Y conferring mutation. The Sanger sequencing of *NA* genes from cases C and D corresponded to the Roche GS FLX generated sequences, and the *NA* master sequences from all cases were identical with the exception of the *NA* H275Y conferring mutation in case D.

**Table 3:** Intra-host variant sites and frequencies in GS FLX sequences from the NA gene in patient D compared to GAIIx sequences

**nt (Residue)**	**Dom Base**	**Freq GS FLX**	**Freq GAIIx**	**Alt Base**	**Freq GS FLX**	**Freq GAIIx**	**Total Coverage GS FLX**	**Total Coverage GAIIx**	**Variation (%) GS FLX**	**Variation (%) GAIIx**
458 (S153N)	G	51	128	A	0	10	51	138	0	7
823 (H275Y)	T	114	134	C	18	4	132	138	86	97

**Footnotes:** ‘nt’ refers to nucleotide; ‘Dom’ refers to dominate base (G = S153, T = 275Y); ‘Freq’ refers to frequency; ‘Alt’ denotes alternative (A = 153N, C = H275).

### Illumina GAIIx sequencing of full genomes

Illumina GAIIx sequencing of cultured samples from cases C and D yielded full genomes covered with a respective average depth of 128 and 127. While 0/137 sequences contained the causative mutation on the *NA* gene at nucleotide 823 in case C, 97% of the sequences in case D (134/138) contained the resistance-conferring mutation (see Table 4). The *NA* gene sequences generated by Sanger, Roche GS FLX and Illumina GAIIx sequencing for cases C and D all corresponded. Master sequences can be found in NCBI for viruses recovered from case C, A/Denmark/524/2009 (accession numbers CY043339-46), and case D, A/Denmark/528/2009 (accession numbers CY043347-54).

**Table 4:** Locations and details of genome nucleotide positions containing variation

**Case**	**Gene**	**nt**	**Dom base (freq)**	**Alt base (freq)**	**Total Cov**	**Var %**	**Log LH (AA)**	**Log LH (AB)**	**Log LH (BB)**	**S or NS.**	**Amino Acid**
C	PA	184	A(116)	C(11)	127	9	−48	−38	−501	NS	I62L
D	PA	1800	T(114)	C(18)	131	14	−77	−39	−524	S	S600
D	HA	844	A(103)	G(33)	136	24	−142	−41	−478	NS	I282V
D	PB1	437	C(106)	T(22)	126	18	−98	−38	−450	NS	T146I
D	PB1	796	C(103)	A(20)	123	16	−86	−37	−430	NS	L266I
D	PB1	1791	C(96)	A(35)	131	27	−149	−39	−407	NS	N597K
D	NA	458	G(128)	A(10)	138	7	−48	−42	−552	NS	S153N
**D**	**NA**	**823**	**T(134)**	**C(4)**	**138**	**97**	**−624**	**−41**	**−17**	**NS**	**H275Y**

**Footnotes: ‘**nt’ refers to nucleotide position. ‘Dom’ refers to dominant nucleotide. ‘Alt’ refers to alternative nucleotide. ‘Cov’ refers to sequence coverage. ‘Freq’ refers to frequency. ‘Var’ refers to level (%) of variant. ‘Log LH’ refers to log likelihood, where AA represents no variation from the reference, BB represents 100% variation from the reference and AB represents some variation from the reference. ‘S’ and ‘NS’ indicates synonymous and non-synonymous amino acid substitutions. The bold text highlights the variation associated with the NA H275Y, oseltamivir-resistance conferring substitution.

Analysis for intra-host variants present in >2% of the sequences across all 8 segments of the influenza genome in cases C and D indicated variation at one, and seven nucleotide positions respectively, including variation at the oseltamivir-resistance conferring mutation in the *NA* gene (Table 4). This variation consisted of five transitions and three transversions and the eight single nucleotide positions were in the *PA*, *HA*, *PBI* and *NA* genes (Table 4). No intra-host variants were seen in the *NS*, *M*, *NP* and *PB2* genes. All variant positions were sequenced with high coverage (a minimum of 123) and high quality (scoring 99 for genotype quality), indicating that they were unlikely to be the result of sequencing errors. Moreover, positions showing variation only contained two possible bases corresponding to either the dominant or the variant sequence, further reducing the possibility of sequencing error. The log-scaled likelihood values supporting intra-host variation (AB) were higher than the support for no variation (AA or BB in Table 4) for all the variant sites, excluding variation at nucleotide position 823 (conferring H275Y substitution) on the *NA* gene. However, given that the 275H genotype was represented in only 3% of the sequences from case D, the log-likelihood value was expected to be low.

Seven of the eight nucleotide positions showing evidence of intra-host variation caused non-synonymous amino acid substitutions, with the synonymous substitution being associated with nucleotide 1800 in the *PA* gene (Table 4). This high level of non-synonymous versus synonymous substitutions is consistent with a previous study [9], where Ghedin *et al*. deep sequenced intra-host variants of an A(H1N1)pdm09 virus and determined that there were 10 variant nucleotide positions causing 10 non-synonymous substitutions.

Five of the eight variant nucleotide positions, inclusive of that encoding H275Y substitution, have been reported previously (based on BLASTn and literature searches at the time of manuscript preparation). A list of the top BLASTn and literature results (limited to the top five) can be found in Table 5. Other than the mutation encoding H275Y substitution, it is unknown whether the other seven intra-host variant nucleotide positions have any effect on the fitness of the virus.

**Table 5:** Top BLASTn and reference hits for intra-host variantsCase

**Case**	**Gene**	**Variant nt Location**	**Reference/Accession number**	**Virus**
C	PA	c	CY018882	A/green wing teal/Ohio/175/1986(H2N1)
			CY080052	A/mallard/Interior Alaska/8BM3519/2008(H12N5)
			CY079192	A/mallard/Interior Alaska/8MP0792R1/2008(H12N5)
			CY079120	A/northern pintail/Interior Alaska/8BM3582/2008(H3N8)
D	PA	1800	CY097775.1	A/mallard/Mississippi/442/2010(H1N1)
			CY062007.1	A/New York/7036/2009(H1N1)
			CY060749.1	A/Ontario/9739/2009(H1N1)
			JN584193.1	A/swine/Quebec/1257774/2010(H3N2)
			CY073799.1	A/duck/Malaysia/2001(H9N2)
D	HA	844	CY061741.1	A/swine/Hong Kong/2314/2009(H1N2)
			Chakrabarti *et al.* (2009)^29^	Dk/India/TR-NIV4396/ 08(H5N1)
D	PB1	437	-	-
D	PB1	796	-	-
D	PB1	1791	-	-
D	NA	458	HM598359.1	A/Thailand/CU-MV56/2010(H1N1)
			Baz *et al.* (2010)^28^	A/Brisbane/59/2007-like(H1N1)
D	NA	823	HM625654.1	A/Rome/PTV8/2009(H1N1)
			HM598322.1	A/Thailand/CU-MV9/2010(H1N1)
			CY092411.1	A/Sydney/DD3-48/2010(H1N1)
			JF807497.1	A/Chennai/10/2009(H1N1)
			CY091665.1	A/Singapore/GP4344/2010(H1N1)

**Footnotes**: BLASTn results for variant nucleotides were only included if they contained 100% identities. ‘nt’ refers to nucleotide.

## Discussion

In this study, case C, who was treated with oseltamivir, most likely infected case D, who was receiving prophylactic doses of oseltamivir. Case C was sampled prior to receiving oseltamivir, while case D was sampled five days after oseltamivir prophylaxis commenced. It has been suggested that resistant viruses carrying the *NA* H275Y substitution suffer no fitness losses compared with those with *NA* 275H wild-type in the absence of drug intervention [12], and since samples were taken from cases A-C prior to oseltamivir-treatment, it is not possible to conclude whether resistance was acquired during their treatment and resistant virus transmitted to case D, or whether resistance emerged during prophylaxis in case D.

However, several pieces of evidence support case C infecting case D (although it cannot be ruled out that the infection originated from case A or B). Firstly, cases A-C tested positive for A(H1N1)pdm09 influenza on June 2^nd^ and they were subsequently isolated. At this time case D remained negative for A(H1N1)pdm09 influenza. Case C and D were in a close, personal relationship and case D had only been in contact with case C (rather than cases A or B) when case D developed symptoms. Secondly, looking to the consensus sequences from patients C and D lends strong support to their epidemiological linkage. For all 8 segments it holds true that the consensus sequences of the virus populations are identical, with the exception of the H275Y conferring mutation in the *NA* gene from case D. Additionally, performing a BLASTn search against the GenBank nucleotide database (online version as of 14/3 2012, http://www.ncbi.nlm.nih.gov/blast) revealed that the *HA*, *NP* and *NS* genes were uniquely identical, meaning that they matched no other sequences in GenBank. The *PB1* sequence matched only a single entry completely, and *PB2* matched only 3 other entries (none of which share a patient origin with the *PB1* hit), leaving only the *PA* and *M* genes matching multiple, globally distributed, entries in the database. For *NA* the sequences derived from patient C also matched multiple hits whereas those encoding resistance in patient D had a single perfect match.

To date, few studies have reported on the intra-host evolutionary dynamics of influenza viruses. One major reason for this has been the limited capacity to detect low frequency virus variants using conventional methods. However, new deep sequencing technologies offer an efficient and economic way to obtain a snapshot of the entire virus population. During this study we demonstrated the diversity between intra-host variants in cases of oseltamivir–sensitive and oseltamavir–resistant A(H1N1)pdm09 infections. While the master sequences of these cases remained identical, but for the mutation encoding the *NA* H275Y substitution, the subpopulations of the virus quasispecies varied significantly. Interestingly, case D contained a mixed population at nucleotide 823 on the *NA* gene, resulting in 3-14% (depending on the sequencing platform used) of the viruses remaining sensitive to oseltamivir. This result is similar to other reports of mixed populations occurring in H275Y variant A(H1N1)pdm09 viruses [9], and together they suggest either that a small proportion of the sensitive viruses survive the treatment, or that the mutation conferring oseltamivir sensitivity rapidly re-establishes itself in the virus population after the bottleneck. These data also suggest that the *NA* 275Y containing virus may be less fit than the sensitive *NA* 275H virus, a hypothesis that is supported by experiments in a ferret model [13], although contradictory to the 275Y variant outgrowing the 275H wild-type in MDCK cells. However, other authors did not find the resistant 275Y virus to be attenuated in other animal models [14]. In order for the oseltamivir-resistant virus to be stable and more fit than the non-resistant counterpart, other changes in the *NA* or *HA* genes may have to be present as has been shown for the former seasonal A(H1N1) virus during 2008 [15].

In addition to harboring a subpopulation of oseltamivir-sensitive viruses, case D was noteworthy for the occurrence of seven additional intra-host variants. Only four of the seven mutations have been reported in previous influenza studies, three of which have been associated with A(H1N1)pdm09 virus (including that conferring H275Y substitution). Firstly, Baz *et al*. [16] reported that a *NA* S153N substitution occurred concomitantly with the H275Y substitution in an oseltamivir-resistant virus. Secondly, the mutation at residue 600 of the *PA* gene appears in numerous consensus sequences submitted to the NCBI database. Thirdly, the mutation conferring *HA* I282V substitution in A(H5N1) influenza virus, was reported by Chakrabarti *et al*. [17], but it was not seen in combination with *NA* H275Y substitution so is unlikely to be linked to oseltamivir-resistance. The occurrence of these intra-host variants in case D may be related to maintenance of virus fitness. However, these mutations occur in non-conserved regions of the influenza A virus genome [18-21], suggesting that they may have arisen as deleterious mutations during a population expansion, with no direct correlation to the NA H275Y conferring mutation.

Given one nucleotide change per 10,000 nucleotides during replication and that most infections are caused by 10–1,000 virions which likely possess varying numbers of nucleotide differences in their genomes, one can expect that each influenza A virion is a possible intra-host variant. However, we identify relatively few variant sites, probably because currently available sequence analysis software do not allow robust intra-host variant analyses and manual curation is necessary. Hence, we believe that with improved bioinformatic tools we would detect more subpopulation variation in our sample set. However, while our levels of intra-host variation seem low, they are consistent with other second-generation sequencing studies. Ghedin *et al*. [9] used a 10% cut-off for including a variant. This conservatively high cut-off ensured that variants were not determined by substitution errors (0.03% per nucleotide on the Roche GS FLX). Using this cut-off, the authors detected only 10 sites containing variation throughout the genome. Similarly Ramakrishnan *et al*. [8] detected between 0 and 10 sites with variation, depending on the sample.

The aim of our study was to characterize the intra-host variation occurring in the virus populations in a transmission chain. There are, however, some limitations to the methodology and processes that can influence the accurate quantification of subpopulation variants: i) cell culturing processes; ii) RT-PCR amplification; iii) emulsion PCR (emPCR) and bridge PCR (in GS FLX and GAIIx sequencing, respectively); and iv) the sequencing process.

Firstly, second-generation sequences for cases C and D were determined from viral culture isolates rather than from clinical specimens directly. It is possible that some of the substitutions observed may have occurred during passage of virus in cells, although reports of genetic variation occurring during MDCK cell passage are predominantly linked to changes in the *NA* and *HA* genes [15]. MDCK cell cultures have been reported to select for new variants, particularly for mutations leading to enhanced growth of virus in these cells [16]. However, given that both cases C and D were cultured in much the same manner, with only one passage more required for case D, they allow direct comparison, with the H275Y conferring mutation being the primary difference between the samples. Additionally, reports of oseltamivir-resistant viruses outgrowing sensitive viruses in MDCK and rhesus monkey kidney cells might suggest that the 3-14% (GAIIx and GS FLX results, respectively) of oseltamivir-sensitive virus that we detected may be an under-estimate of persisting oseltamivir-sensitive viruses in the case D patient [9,16]. Thus, a direct analysis of clinical specimens for the polymorphisms at the sites listed in Table 3 would be required to confirm these observations. However, due to the low virus concentration in the case C and D clinical samples, we were unable to convert the uncultured samples into libraries. Future generations of sequencing platforms, such as the PacBio RS, requiring lower starting amounts of template and the ability to sequence single influenza segments may resolve these issues.

Secondly, PCR-induced errors were minimized in the preparation of the One-Step RT-PCRs and library amplification steps by using Platinum and Phusion (respectively), both high fidelity polymerases. Further, for the PCR replication step (5 separate reactions for the Illumina GAIIx library template), a small number of amplification cycles (18 cycles total) were used in the preparation of the amplified Illumina GAIIx library.

However, despite the aforementioned precautions, evidence of amplification biases were still evident in the variant sites detected in the *NA* gene of case D. The H275Y conferring mutation was present in 86% and 97% of sequences using the GS FLX and GAIIx platforms, respectively. This difference is likely to represent biases introduced during RT-PCR or emPCR/bridge PCR, rather than during cell culturing given that these libraries were constructed based on the same cell culture products. Repeating/duplicating the sequencing process could resolve this discrepancy. It should be noted that the GAIIx library was built using 5 replicate RT-PCR reactions; hence this could represent a more reliable variant frequency measurement. The differences could also represent biases introduced due to the differences in GS FLX and GAIIx library amplification and sequencing methods.

Moreover, we detected the *NA* S153N conferring mutation in 7% of the sequences obtained on the GAIIx platform but not those from the GS FLX platform. This could be explained by: i) a nearly 3-fold decrease in coverage of this site using the GS FLX platform (51-fold coverage compared to 138-fold coverage on the GAIIx platform); ii) the S153N conferring mutation arose as a sequencing error on the GAIIx sequencer; or iii) preferential PCR or sequencing bias in the GS FLX library for the dominant 153S encoding sequences. Given that the S153N encoding variant was present in 10/138 sequences, post-filtering, this anomaly is most likely explained by hypotheses i) or iii).

Given that oseltamivir treatment would cause a virus bottleneck, one might expect to see a reduction in the genetic diversity of the virus population [22]. However, this is not what was observed in case D, as was seen in another A(H1N1)pdm09 study [9]. Specifically, Ghedin *et al*. [9] compared virus sequences in specimens taken from an individual prior to and after the emergence of oseltamivir-resistance and detected no variation in the oseltamivir-sensitive sample, compared to 10 nucleotide positions showing variation in the resistant sample. There were no common positions of nucleotide variation, excluding the H275Y conferring mutation, between the resistant virus specimens analyzed in the two studies.

How might one explain these observations? Previously it was shown that RNA viruses can undergo rapid evolution following bottleneck events, leading to the accumulation of neutral and deleterious mutations and, rarely, the emergence of biologically fit variants [23,24]. Therefore, in the present study, it would appear that the introduction of oseltamivir either as treatment to case C or as prophylaxis to case D created selective pressure that caused the drug-resistant viruses to rapidly accumulate in case D. However, as we had only one collection date for each case in this study, we could not determine whether or not the levels of virus variants remained constant throughout the infections. For future studies of intra-host variation it will be interesting to investigate samples taken over time-series, both within single infections to monitor the variation within an individual, and within longer transmission chains. The latter will enable characterization of whether the unexpected diversity post-bottleneck is maintained, or lost.

In conclusion, we used deep sequencing to characterize the diversity of influenza viruses within a transmission chain that involved the development of oseltamivir resistance. As in previous studies, we detected the presence of low levels of oseltamivir-sensitive virus in the case harboring oseltamivir-resistant viruses, suggesting either survival through the treatment bottleneck, or rapid re-evolution of the sensitivity-conferring mutation post bottleneck. In addition, we paradoxically detected higher levels of intra-host variation in the case harboring oseltamivir-resistant virus, compared to the case carrying oseltamivir-sensitive virus. This contributes evidence to support the hypothesis that post-bottleneck, virus population expansions result in the generation of greater levels of genetic diversity than are otherwise found in the population.

## Materials and methods

### Ethics approval

Research carried out on humans was in compliance with the Helsinki Declaration. Following the guidelines of the National Board of Health (Denmark), informed consent for use of the sequences in this paper was not required from the sample sources, as the influenza viruses were analyzed for clinical diagnosis under Danish Data Protection Agency (Datatilsynet) permit number 2007-54-0364 provided to Dr Lars P. Nielsen, State Serums Institute. As required by this agreement, the identity of the patients remains anonymous.

### Consent

Written informed consent was obtained from the patient for publication of this report and any accompanying images.

### Virus isolation and extraction

Throat swabs collected from cases A-D and contacts were suspended in 1 ml of virus transport medium, 200 μl of which was used for extraction of viral RNA by a MagNA Pure LC Instrument applying the MagNa Pure LC Total Nucleic Acid Isolation Kit (Roche diagnostics, Basel, Switzerland).

### RT-PCR diagnosis

Initial A(H1N1)pdm09 diagnosis was performed by real-time RT-PCR. Extracted RNA (5 μl) was added to 20 μl of master mix consisting of 10 μM of each primer and 2 μM of the Taqman probe together with reagents from the OneStep® RT-PCR Kit (QIAGEN, Hilden, Germany) according to the manufacturer’s instructions. Primer sequences are available upon request. Target sequences were amplified on the MX3005 Thermocycler system (Stratagene, CA, USA) with the following thermal cycling parameters: 20 minutes at 50°C, 15 minutes at 95°C followed by 45 cycles of 15 seconds at 95°C and 60 seconds at 55°C.

### Sanger sequencing

The *NA* genes from patient samples for cases C and D, prior to cell culturing, were amplified by traditional one-step RT-PCR and Sanger sequenced as previously described [25]. We were unable to amplify all other gene segments from cases C and D prior to cell culturing.

### Inoculation and neuraminidase inhibition assay

Patient samples from cases C and D were inoculated onto primary swine kidney cells (due to slow growth in MDCK cells) and were further propagated on MDCK cells and in 10-days-old fertilized chicken eggs. Specifically, sample C was passaged three times in swine kidney cells (titre 4 × 10^5^ TCID50/ml), while sample D was passage four times in the same manner. The viruses were harvested and examined by neuraminidase inhibition assay to determine the NI drug sensitivity phenotype, at the WHO Collaborating Centre for Reference and Research on Influenza, London, UK. Inhibition of neuraminidase activity was measured using the fluorescent substrate, 2’-(4-methylumbelliferyl)-α-D-N-acetylneuraminic acid (MUNANA; Sigma). Briefly, 20 μl of virus was incubated with 30 μl of 100 μM MUNANA in 32.5 mM MES buffer pH 6.5 containing 4 mM CaCl_2_ for 1 hr at 37°C. The reaction was stopped by addition of 150 μl 0.1 M glycine containing 25% ethanol and 2% SuperQ pH 10.7 and fluorescence of the released 4-methylumbelliferone was measured at excitation and emission wavelengths of 360 nm and 460 nm, respectively. The activity of each virus sample was titrated, by assaying serial twofold dilutions, and the amount of virus adjusted to equivalent NA activities, which fell in the linear portion of the activity curve. Each virus was preincubated for 30 minutes at 37°C with oseltamivir or zanamivir at final concentrations of 4 μM-15.3pM, in serial 4-fold dilutions, NA activity measured and the drug concentration that inhibited 50% of the neuraminidase activity (IC_50_) was determined [26].

### Deep sequencing

#### Roche Genome Sequencer FLX

The level of influenza RNA in the samples from cases A and B was of insufficient quantity/quality to enable complete influenza genome sequencing using universal influenza primers that co-amplify all 8 segments in a single RT-PCR reaction [27]. Hence, Center for Disease Control (CDC) primers were used to RT-PCR amplify the *NA* genes from cases A, B (both patient samples), C and D (both cultured samples) [28]. Although it would have been preferable to obtain sequence from the uncultured patient samples for cases C and D, to allow assessment of potential selection of sequence variants during the culture process, we were unable to construct libraries on these samples, hence the libraries were based on the cultured samples. PCR products were converted into Roche GS FLX libraries and were sequenced on 1/8^th^ of a PicoTitre Plate (PTP), using LR70 chemistry, following the manufacturer’s protocol (Roche, Basel, Switzerland).

#### Illumina GAIIx

An Illumina GAIIx was used for full influenza genome sequencing (all 8 gene segments) for cell-cultured virus from cases C and D. In comparison to the Roche platform, the Illumina GAIIx yields several orders of magnitude more sequences in a single sequencing run, thereby allowing the generation of deeper sequencing coverage of the influenza genomes. Library building and sequencing of samples were performed using a previously described method [29]. Briefly, full influenza genomes sampled from cases C and D were converted into cDNA and amplified, in five separate SuperScript III One-Step RT-PCR reactions (with Platinum Taq High Fidelity: Invitrogen, Carlsbad, CA) reactions, using a previously published method [27]. The temperature cycle parameters were 94°C for 30 sec, 45°C for 30 sec, and 68°C for 3 min, followed by 31 cycles at 94°C for 30 sec, 57°C for 30 sec, and 68°C for 3 min. There were two reasons for amplifying the samples in five separate RT-PCR reactions: firstly, to create a large volume of sample that could then be concentrated; and secondly, to reduce the effects of biases caused by RT-PCR amplification. The five RT-PCR reactions were then pooled and built into indexed Illumina GAIIx libraries using the NEBNext Quick DNA Sample Prep Master Mix Set 2 (New England Biolabs, Ipswich, MA, USA) and sequenced on 1 lane of an Illumina GAIIx following the manufacturer’s protocol (Illumina, San Diego, CA, USA) as part of a larger pool of other libraries not relevant to this study.

### Data analysis

#### Roche Genome Sequencer FLX

Sequence and quality files produced after sequencing were manipulated using R scripts in order to obtain fastq files suitable for subsequent processes. Roche GS FLX reads were mapped to the Influenza A(H1N1)pdm09 prototype, A/California/07/2009, *NA* gene (GI:229396468) using the bwasw algorithm [30] included in the bwa v.0.5.9-r16 package: bwasw was preferred over the regular bwa due to the length of sequence reads. Resulting bam files were filtered for reads with two or more different mapping locations and chimeric reads (defined as having two or more different mapping locations for different sections in the read) for the following analyses, using Perl scripts. After filtering, samtools v.0.1.14 [31] rmdup was used in order to collapse PCR duplicates. PCR duplicates are defined as reads with the same 5’ mapping position and orientation; only the read with the highest mapping quality is kept. Samtools ’mpileup’, with its default parameters, was used on the sorted and collapsed alignments in order to produce a base pileup. Using the output from ’mpileup’, genotypes for each position were called using bcftools which implements a Bayesian diploid SNP calling model, based on base and mapping qualities. This model is useful when attempting to characterize the intra-host diversity due to its ‘calling of sites with variation’ capability.

#### Illumina GAIIx

Post Illumina GAIIx sequencing, data was sorted using the method detailed in Kampmann *et al.*[29]. Briefly, reads were aligned to the reference A/California/07/2009 sequences using bwa v.0.5.9-r16 [30]. The bam files were manipulated using Perl scripts, along with samtools v.0.1.14 [31], and GATK v.1.0.4641 M [32]. Coverage and depth of coverage was calculated with GATK’s DepthOfCoverage analysis. Variants were called using GATK’s UnifiedGenotyper walker [33]. The resulting vcf files were manually sorted to remove variants that occurred only once (i.e. singletons) or at a frequency of less than 2% of bases at the specific location.

## Competing interests

The authors declare that they have no competing interests.

## Authors’ contributions

MTPG, LPN, KB, and SLF were responsible for the conception and design of the project, and drafting of the manuscript. KB, LPN, SLF and MLK conducted laboratory-based experimental work. SSP and TGJ made the initial virus detections and collected clinical data. RD and AH propagated viruses for phenotypic characterization, performed independent Sanger sequence analyses and assisted with manuscript revision. Sequencing facilities were provided by EW. JVMM, MCAA, CAWB and SLF were responsible for data analysis. All authors read and approved the final manuscript.

## Ethics approval

Following the guidelines of the National Board of Health (Denmark), informed consent for use of the sequences in this paper was not required from the sample sources, as the influenza viruses were analyzed for clinical diagnosis under Danish Data Protection Agency (Datatilsynet) permit number 2007-54-0364 provided to Dr Lars P. Nielsen, State Serums Institute. As required by this agreement, the identity of the patients remains anonymous.
